# Analysis of Superjunction MOSFET (CoolMOS™) Concept Limitations—Part I: Theory

**DOI:** 10.3390/ma18235451

**Published:** 2025-12-03

**Authors:** Zbigniew Lisik, Jacek Podgórski

**Affiliations:** Department of Semiconductor and Optoelectronic Devices, Lodz University of Technology, Aleje Politechniki 8, 93-590 Lodz, Poland

**Keywords:** MOS devices, power MOSFETs, superjunction, power semiconductor devices, power electronics, device performance optimization

## Abstract

The CoolMOS™ (Infineon Technologies AG, Munich, Germany) has been considered a device that alleviates high-voltage limitations of unipolar power devices, but although the theoretical considerations seem to confirm such a possibility, this expectation has not been fulfilled until now. This paper identifies limitations of the CoolMOS™ concept. The analysis was carried out in two steps. The first step aimed at the theory of high-voltage superjunction and its implementation into a power VDMOS transistor, which resulted in the modified construction called CoolMOS™. The investigations have shown that the superjunction effect is not an inherent feature of high voltage junctions formed as a characteristic meander-like p-n junction. Such a junction starts to work in SuperJunction Mode (SJM) just when the electric field strength reaches the magnitude of the threshold electric field E_th_. Also, other theoretical constraints concerning the SJ diode and CoolMOS™ design have been presented. The second step aimed at the physical and technological limitations that have been identified, taking advantage of numerical investigations for CoolMOS™ structures developed on the basis of a typical VDMOS one.

## 1. Introduction

**Figure 1:**
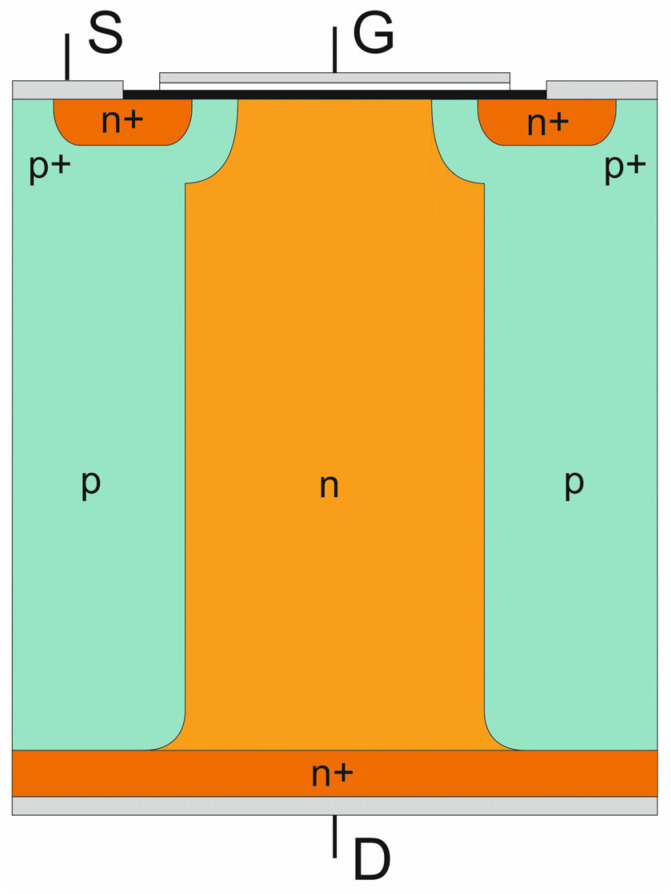
Basic cell of CoolMOS™ transistor.

The concept of the CoolMOS™ transistor is based on the conventional MOSFET principle that has been supplemented by the superjunction idea used for the reconstruction of the high voltage junction [1,2]. The main disadvantage of the conventional VDMOS (Vertical Double Diffusion MOS), limiting its high voltage applications, consists of the presence of a low-doped n-layer. Its large width is necessary to meet high blocking voltages, but it leads to an unacceptably high forward voltage. The CoolMOS™ design was created to solve this problem by reconstructing the high voltage junction. Now, its cell, shown in Figure 1, consists of one central n-doped column surrounded by two p-doped ones characterized by the same doping level, and the plane of the high voltage junction is partially vertical instead of being horizontal only.

**Figure 2:**
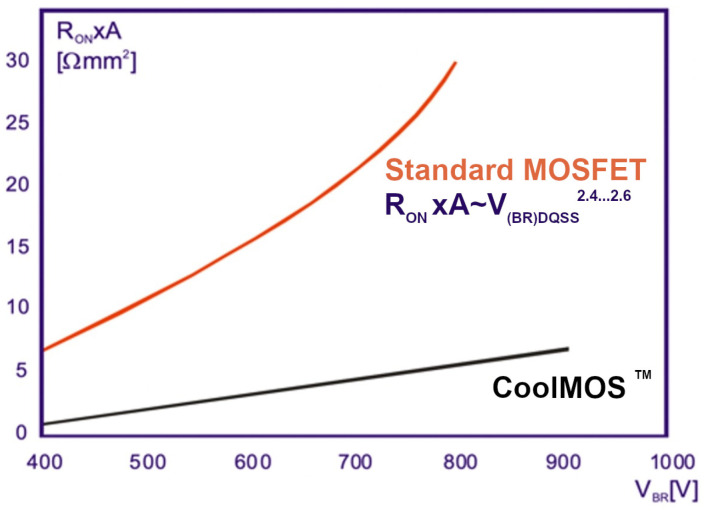
Comparison of theoretical relations between on-resistance R_on_xA and blocking voltage V_BR_ presented in [2] for standard MOSFET and CoolMOS™, respectively.

As a result, the direction of the electric field is also partially horizontal, the electric field can be spread evenly over both columns, and when the thickness of the columns is not too large, the superjunction effect occurs. It means that at the same blocking voltage, the doping of the n-layer, conducting current towards the drain, can be larger, which results in the lower transistor on-resistance. The prospective advantage of the CoolMOS™ construction over the VDMOS one is presented in many publications [3,4] in a highly convincing manner and is shown in Figure 2.

Though the CoolMOS™ idea was very promising, the rating parameters of the first devices introduced to the market in 1999 were not as attractive as one could expect. Since that time, their construction has been the subject of many investigations both theoretical, using simple analytical approaches [5,6,7,8,9,10,11] and equivalent circuit models [12,13], and numerical ones [14,15,16,17], dealing with particular aspects of superjunction application, as well as experimental works [18,19,20,21], resulting in new solutions, covering more intricate design, e.g., the one presented in overview paper [22]. However, until now, their maximum blocking voltage has not exceeded 800 V. One can suppose that it results from the fact that, although the CoolMOS™ basics are well known, the phenomena taking place inside manufactured devices limit their real possibilities. Unfortunately, this problem has so far only been addressed in the literature and has not been the subject of serious published investigations. This paper aims to better understand these phenomena, especially with respect to their run in the vicinity of any fabrication divergences in comparison to the CoolMOS™ concept. Part I is concentrated on the theoretical considerations aimed at identifying any physical constraints in the superjunction concept, whereas Part II of this paper gathers the results of a numerical investigation that takes advantage of Sentaurus TCAD (Synopsys Inc. Mountain View, CA, USA) [23], dealing with the influence of the divergences between the concept of ideal superjunction and the technology limitations on the real device work.

The theory of p-n superjunction has been outlined in [1,2], where some theoretical formulas were presented, but they were not extended to the phenomena in an ideal p-i-n diode, being the origin of its concept. This was conducted in Section II, dealing with the superjunction origin and resulting constraints. The solution was the subject of several numerical investigations [6,24], and it was applied in many manufactured device structures with the CoolMOS™ as the well-known commercial representative. However, nowhere have the constraints and consequences of the superjunction design been clearly taken out of the considered formulas and explicitly presented. Such knowledge is necessary when the limitations of the superjunction application are to be considered. It is presented below with respect to the specificity of CoolMOS™ design.

## 2. Materials

### 2.1. Superjunction Origin

**Figure 3:**
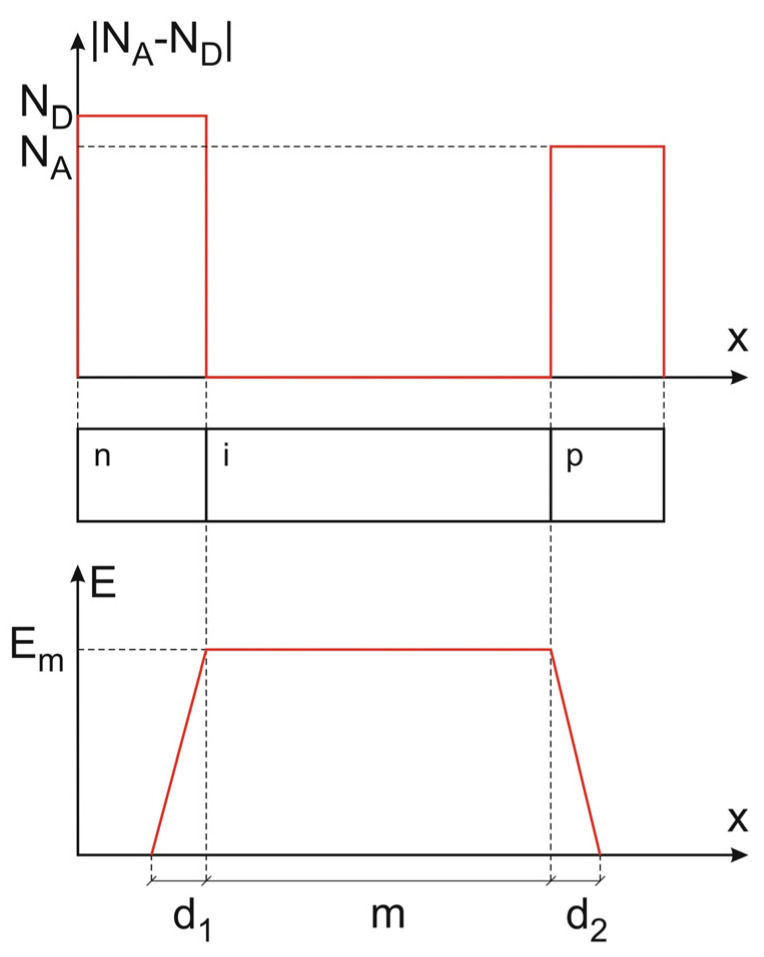
Ideal p-i-n diode: dopant concentration (**upper**) and electric field distribution (**down**).

As the origin of the superjunction concept, one can consider the ideal p-i-n diode structure depicted in Figure 3. Its middle layer has no dominant dopants, so a no-space charge region can build up in it, which results in the constant electric field strength, E_m_, in the layer at the reverse bias condition. The changes in the electric field take place in the border n- and p-layers only, and the voltage drop V_D_ over the whole reverse-biased diode is described by (1), where q is the electron charge, and ϵ is the material permittivity.

(1) $$V_{D}=V_{d1}+V_{m}+V_{d2}=\frac{\epsilon E_{m}^{2}}{2qN_{D}}+mE_{m}+\frac{\epsilon E_{m}^{2}}{2qN_{A}}=\left(m+\frac{\epsilon }{2}\left(\frac{1}{N_{D}}+\frac{1}{N_{A}}\right)E_{m}\right)E_{m}$$

According to (1), the breakdown voltage of such an ideal p-i-n diode has no limits, since by keeping the magnitude E_m_ below the electric field strength E_crit_, initiating the avalanche breakdown, one can achieve a safe blocking voltage just by increasing the width m of the middle layer that acts as the “no-charge flat field domain” (nc-FFD). Although the concept of the p-i-n diode with unlimited breakdown voltage is very exciting, unfortunately, it also has two significant weaknesses that definitely eliminate its application. Firstly, obtaining an ideal intrinsic semiconductor layer is very difficult; secondly, such a layer will have very large resistivity, leading to an unaccepted large diode on-resistance. Nevertheless, it shows that it is possible to design high voltage devices with the blocking voltage far beyond the limits determined by the critical electric field E_crit_ [14,25]. It demands only to design the device in such a way that under the reverse bias conditions, an FFD layer with a flat electric field distribution along the voltage drop occurs.

In the case of an ideal p-i-n diode, the above condition is fulfilled by the i-layer that is completely free from any uncompensated built-in charge, particularly, from any dopants. Generally, it is impossible in doped materials, in which the presence of uncompensated charge (ions) results in a variation in the electric field. However, there are some exceptions to the above rule, when in some direction, no variation in electric field strength in the area with uncompensated charge can occur. One can distinguish between the two such situations presented below.

#### 2.1.1. Built-In Charge Flat Field Domain (bcFFD)

**Figure 4:**
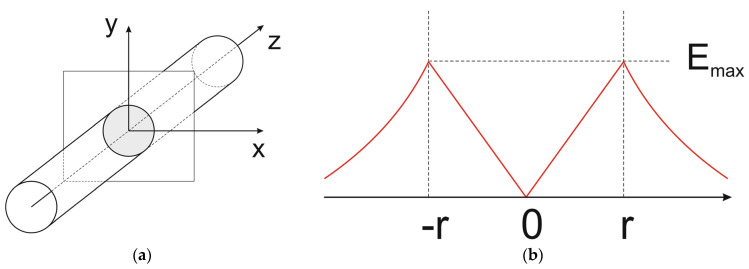
Infinite cylinder covering homogeneous charge distribution (**a**) and corresponding electric field distribution along the *x*-axis in the sketched cross-section perpendicular to the *z*-axis (**b**).

**Figure 5:**
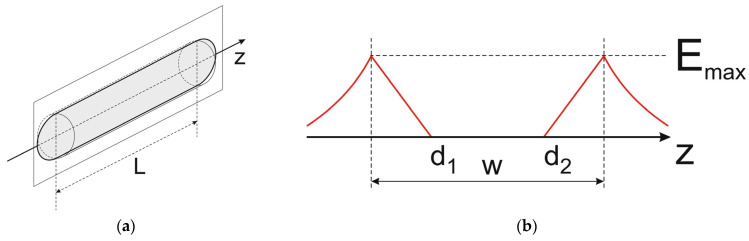
Terminated cylinder covering homogeneous charge distribution (**a**) and corresponding electric field distribution along *z*-axis (**b**).

The situation takes place, e.g., in the infinite cylinder shown in Figure 4a with a homogeneous charge distribution. In any cross-section of the cylinder perpendicular to the *z*-axis, the same radially symmetrical electric field distribution with its maximal magnitude E_max_ on the cylinder surface shown in Figure 4b occurs, whereas the electric field strength along the *z*-axis is constant and, in particular, equal to zero on the *z*-axis. If the cylinder is terminated, e.g., by two semi-spheres like in Figure 5, the electric field distribution inside the cylinder changes a little, but in the central part of the cylinder, the region with constant electric field along the *z*-axis remains unchanged.

It means that when the external voltage drop between the semi-spheres is applied, the central part of the cylinder behaves as an FFD layer like the i-layer in a p-i-n diode in Figure 3. The only difference consists of the fact that, in the p-i-n diode, the constant magnitude of the electric field E_m_ is at any point of the i-layer, whereas in the cylinder, each line parallel to the *z*-axis has its own constant magnitude of electric field.

#### 2.1.2. Doped Semiconductor Flat Field Domain (dsFFD)

**Figure 6:**
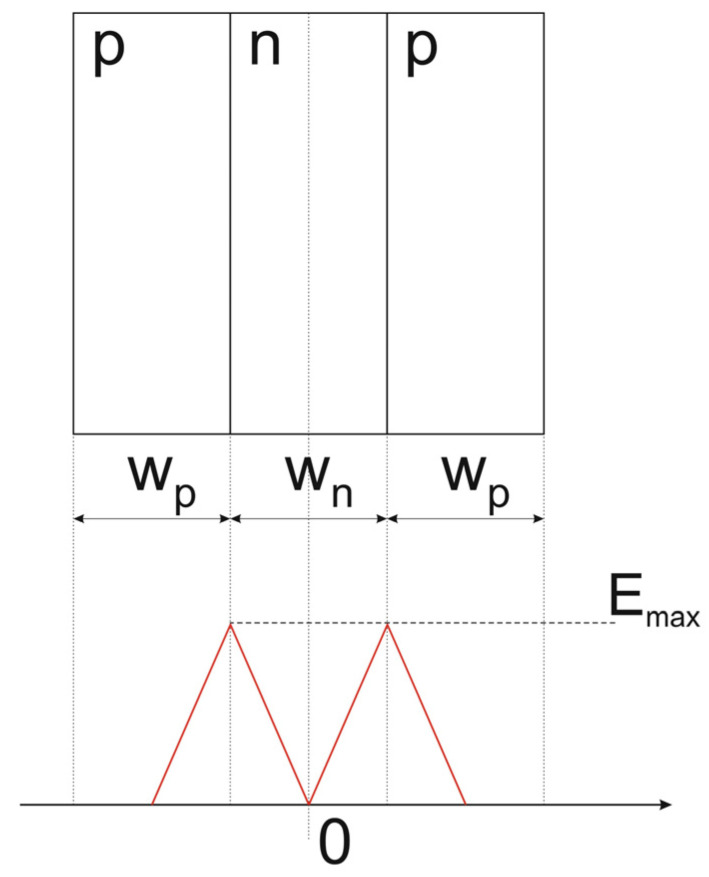
N-type dsFFD layer formed by two parallel symmetric p-n junctions.

In doped semiconductors, the dsFFD, similar to the bcFFD shown in Figure 4, may be composed of two symmetric parallel p-n junctions having one common layer, e.g., an n-layer, as it is depicted in Figure 6. Its thickness w_n_ is fixed to ensure that their SCRs (space charge regions) meet in the center, filling in the middle layer completely when the junctions are unbiased. As in the case of the cylinder in Figure 4, at the junction planes limiting the middle layer, the electrical field strength is maximal E_max_, whereas inside the center (x = 0), its component along the junction planes is equal to zero. Depending on the type of middle layer, one can distinguish n- or p-type dsFFD. The dsFFD phenomenon can be employed to create a layer with a flat electric field distribution, necessary to obtain a high-voltage diode, and it is the basis for the superjunction concept. Note that the dsFDD phenomenon has a one-dimensional character and takes place only in a homogeneous doped domain limited by two parallel junctions. It allows to achievement of the flat component of the electrical field in the depletion area along lines parallel to the junction planes, when E_max_ is lower than the electric field strength E_crit_. In Figure 6, the depletion area covers the whole middle layer.

### 2.2. Superjunction Diode

**Figure 7:**
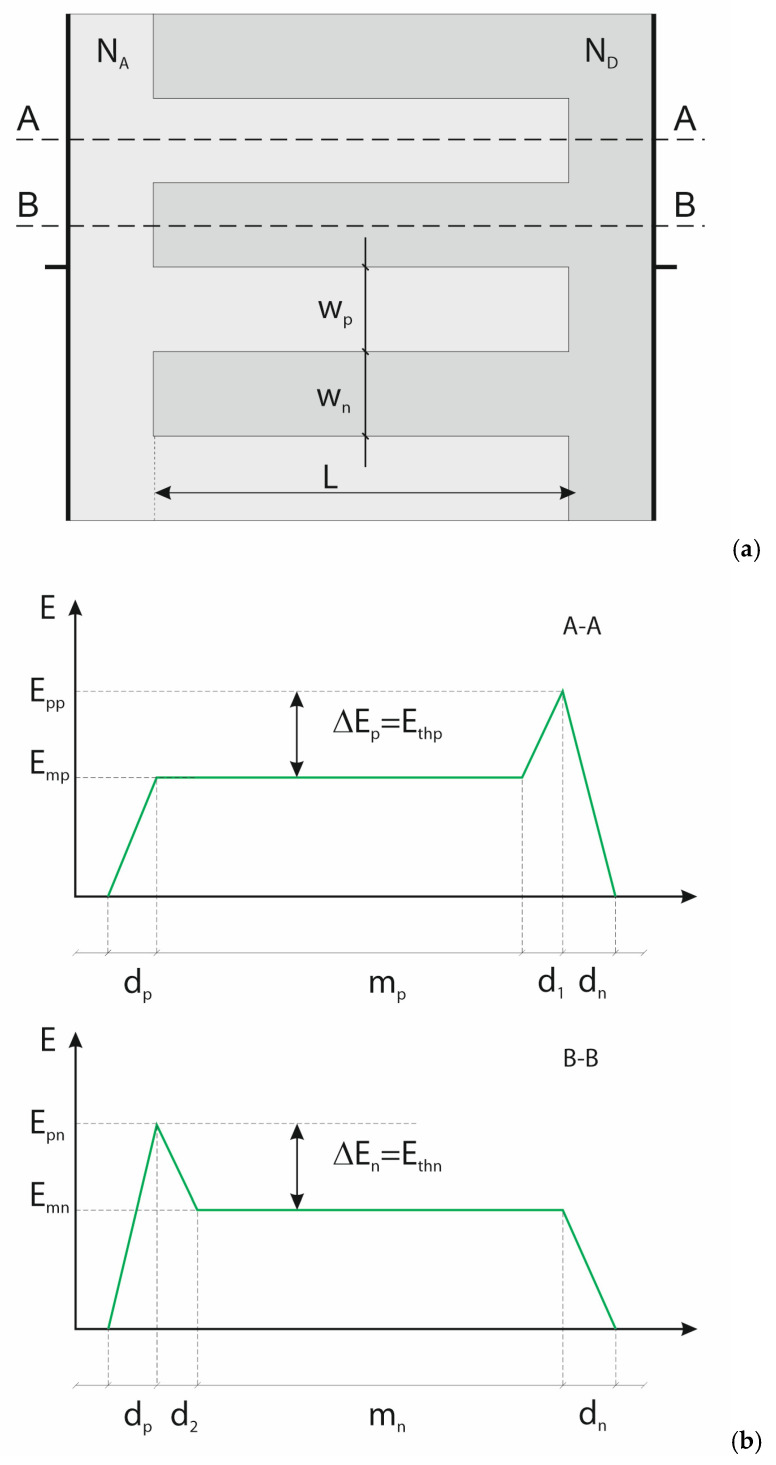
Sketch of superjunction structure (**a**) and the distribution of electric field along of axis A–A and B–B in reverse biased superjunction (**b**).

The layers exhibiting the n- and p-type dsFFD ability are the bricks for a superjunction that is formed as a set of parallel pillars of the same length, n- and p-type by turns, terminated by n- and p-contact layers. It leads to the characteristic meander-like p-n junction shown in Figure 7a. The width of the pillars need not be the same but should be equal to or larger than the width of the middle layer in Figure 6, in which the dsFFD is built-in. In this case, the FFD appears just after the application of reverse bias. In the superjunction, each pillar acts as a separate p-i-n diode, from Figure 3, and can be analyzed separately.

#### 2.2.1. Reverse Bias Ability

Generally, one can distinguish two stages in superjunction behavior under the reverse bias increase. The first represents the Normal Node (NM), when the electric field spreads over the pillars area, its strength is perpendicular to the junction plane, and no dsFFD area occurs. It finishes when the whole pillar areas are occupied by the electric field and the SCRs of opposite junctions meet in the pillar center, as in Figure 6. At this moment, the dsFFD area appears, the magnitude of the electric field strength at any point of the p-n junction plane is the same and defined as the threshold electric field E_thn_ in n-pillar or E_thp_ in p-pillar. Now, when the magnitudes of threshold electric fields are lower than the electric field strength corresponding to the avalanche breakdown E_crit_, the second stage, representing the SuperJunction Mode (SJM), starts to develop, like in the case of a p-i-n diode in Figure 3. In the SJM, due to the superjunction phenomenon, the electric field increases inside both pillars, keeping its flat distribution of magnitude E_mn_ and E_mp_, respectively. It is shown in Figure 7b, presenting the electric field distribution at the SJM conditions in the planes A–A and B–B located in the middle of the p- and n-pillar, where d_1_ = 0.5w_p_ and d_2_ = 0.5w_n_.

Taking into account the Poisson equation, one can evaluate the reverse voltage drop V_SJp_ along the A–A axis:

(2) $$V_{{SJ}_{p}}=V_{dn}+V_{d1}+V_{m}+V_{dp}\;=\frac{E_{pp}d_{n}}{2}+\frac{∆E_{p}w_{p}}{4}+\frac{E_{mp}w_{p}}{2}+E_{mp}m_{p}+\frac{E_{mp}w_{p}}{2}\;=\frac{E_{pp}d_{n}}{2}+\frac{∆E_{p}w_{p}}{4}+\frac{E_{mp}w_{p}}{2}+\left(E_{pp}-E_{thp}\right)m_{p}+\frac{E_{mp}w_{p}}{2}$$

Taking advantage of the relations:

(3) $$d_{n}=\frac{{\epsilon E}_{pp}}{qN_{D}}$$

(4) $$d_{p}=\frac{{\epsilon E}_{mp}}{qN_{A}}$$

(5) $$\frac{w_{p}}{2}=\frac{\epsilon {∆E}_{p}}{qN_{A}}$$

one can simply transform (2) into:

(6) $$V_{{SJ}_{p}}=\frac{\epsilon E_{pp}^{2}}{2q}\left(\frac{1}{N_{D}}+\frac{1}{N_{A}}\right)+\left(E_{pp}-E_{thp}\right)m_{p}$$

Similar considerations, performed for the n-pillar along the axis B–B in Figure 7, lead to a similar expression for the reverse voltage drop V_SJn_ over the considered superjunction structure:

(7) $$V_{{SJ}_{n}}=\frac{\epsilon E_{pn}^{2}}{2q}\left(\frac{1}{N_{D}}+\frac{1}{N_{A}}\right)+\left(E_{np}-E_{thn}\right)m_{n}$$

**Figure 8:**
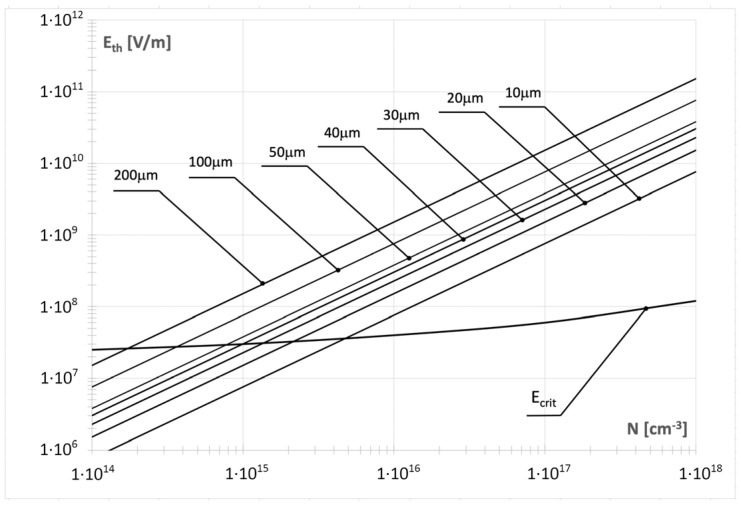
Influence of dopant concentration on the threshold electric field strength E_th_ for different magnitudes of the width w and on the critical electric field strength E_crit_(N).

The derivation presented above of the Formulas (6) and (7) allows presenting very clearly the crux of the ideal superjunction concept in several items below:

- Comparing (6), (7), and (1), one can notice that they are almost identical. They describe the same FFD phenomenon taking place in the diodes corresponding to p- and n-pillars of the superjunction diode in Figure 7 and the ideal p-i-n diode in Figure 3. Its presence leads to devices with generally unlimited blocking voltage. In the case of diodes corresponding to pillars in Figure 7, they can be formally considered as separate devices, although eventually they are aggregated into one device, which results in the identical length L and the identical maximal electrical field strength E_pp_ = E_pn_. In the paper, the p-pillar diode is considered the main one, and next, the results are extended to the n-pillar diode.
- According to (6) and (7), the reverse voltage drops, V_SJp_ and V_SJn_, increase with the increase of m_n_ and m_p_, whereas the maximum electric fields, E_pp_ and E_pn_ remain unchanged. For a symmetrical superjunction, in which N_A_ = N_D_, one can simplify Equations (6) and (7), taking into account that d_1_ = d_2_, and m_n_ = m_p_ = *m*. Now they become identical: V_SJp_ = V_SJn_ = V_SJ_.
- In the parallel pillar connection, the relation *V_SJn_* = V_SJp_ must be kept at all times. Since the electric field strength on the junction plane is constant, the relation E_pp_ = E_pn_ is fulfilled. To obtain E_thn_ = E_thp_ = E_th_, the construction of the diode must ensure that the dsFFD areas come out in both the pillars at the same bias.
- In the pillars, the avalanche breakdown occurs when the maximum electric field, E_pp_ or E_pn_, reaches the local critical magnitude E_critp_ or E_critn_, which may locally differ, e.g., due to the changes in dopant concentration, as it is shown in Figure 8. So, the breakdown voltage for p- or n-pillar is:

(8) $$V_{B_{p}}=\frac{\epsilon E_{{crit}_{p}}^{2}}{2q}\left(\frac{1}{N_{D}}+\frac{1}{N_{A}}\right)+\left(E_{{crit}_{p}}-E_{th}\right)m_{p}$$

(9) $$V_{B_{n}}=\frac{\epsilon E_{{crit}_{n}}^{2}}{2q}\left(\frac{1}{N_{D}}+\frac{1}{N_{A}}\right)+\left(E_{{crit}_{n}}-E_{th}\right)m_{n}$$

- The first element of Relations (8) and (9) corresponds to the breakdown voltage of a planar p-n junction, whereas the second one represents the complementary component introduced by the superjunction effect. It does not occur when E_th_ ≥ E_crit,_ and the breakdown voltage is defined by the doping concentrations, N_A_ and N_D_ only. The superjunction component in (8) and (9) can be presented generally as:
  (10) $$V_{BSJ}=\left(E_{crit}-E_{th}\right)m=E_{crit}\left(1-\frac{E_{th}}{E_{crit}}\right)m\;V_{BSJ}=E_{crit}\left(1-\gamma \right)m$$
  where γ = E_th_/E_crit_ ∈ <0;1>—the superjunction coefficient.

- Introducing ∆E_p_ = E_th_ < E_critp_ into (5), one can obtain the design condition ensuring the presence of the superjunction effect in the p-pillar:

(11) $$N_{A}w_{p}<\frac{2\epsilon E_{{crit}_{p}}}{q}$$

- Similar consideration performed for the n-pillar will result in a similar design condition, ensuring the presence of the superjunction effect in the n-pillar:

(12) $$N_{D}w_{n}<\frac{2\epsilon E_{{crit}_{n}}}{q}$$

- In Figure 7, the superjunction is formed as a parallel connection of several p- and n-pillars with their own breakdown voltages, V_Bp_ and V_Bn_, respectively. Generally, they may be different when E_critn_ ≠ E_critp_ and the total superjunction breakdown voltage is equal to the lower pillar breakdown voltage V_Bp_ or V_Bn_.

Introducing E_thp_ instead of ∆E_p_ into (5), one can obtain the relation between the threshold voltage E_thp_ and the p-pillar width w_p_ and doping density N_A_. One can also obtain exactly the same relation for the n-pillar, which will cover the threshold voltage E_thn_, the width w_n_, and the doping density N_D_. Since both relations are identical, one can consider them jointly using their general representation (13).

(13) $$E_{th}=\frac{qNw}{2\epsilon }$$

This analysis has been illustrated in Figure 8, where a set of linear characteristics E_th_(N) for constant values of w was compared with the curve E_crit_(N), which represents the dependence of the critical electric field strength on the dopant concentration in silicon [13]. Each intersection point between the E_crit_(N) curve and the linear characteristics E_th_(N) corresponding to a specific width w defines a condition in which the threshold electric field equals the critical electric field, E_th_ = E_crit_(N). At these points, the superjunction structure reaches its maximum efficiency, and the superjunction coefficient attains its highest value, γ = 1. In other words, these intersections mark the combinations of dopant concentration N and width w for which the superjunction effect fully contributes to the breakdown voltage, representing the ideal design condition for maximizing the performance of superjunction diodes.

**Figure 9:**
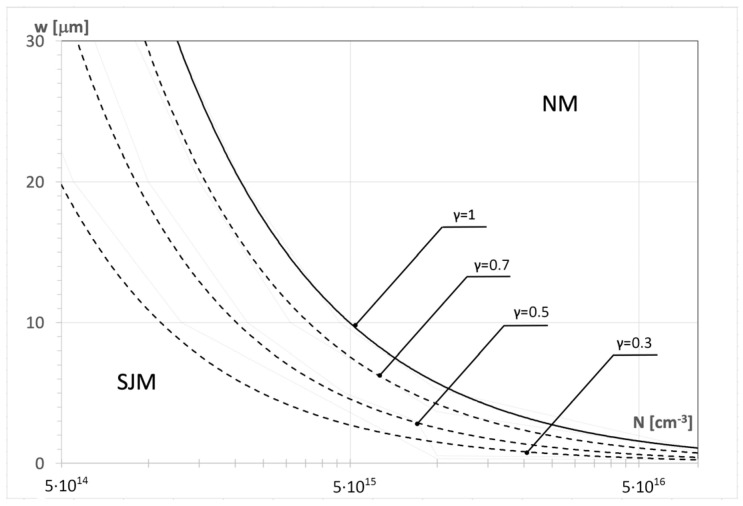
Definition of the superjunction mode (SJM) area in the (N,w) domain. The area for particular γ is below the corresponding curve (dashed line).

According to (10), it means that at the corresponding couples (N,w), the SJ electric field distribution already occurs, but corresponding to its superjunction compound V_BSJ_ = 0. It is shown in a more useful way in Figure 9, which covers the curve presenting the relation between the dopant concentration N and the width w for γ = 1 (solid line). It divides the (N,w) domain into two parts. The bottom area, signed as SJM, gathers all the points (N,w) ensuring the design of diodes that can work in the superjunction mode with the superjunction component V_BSJ_ > 0, whereas the upper area gathers the points corresponding to the diodes that, having the characteristic meander-like p-n junction shown in Figure 7a, can work in the normal mode only (NM).

Figure 9 can be an efficient tool supporting the SJ diode design when it is replenished by more curves drawn for a lower superjunction coefficient γ, as has been performed. Each curve in the figure corresponds to a different value of the coefficient γ, which determines the degree of superjunction contribution to the breakdown voltage. The curve for γ = 1 (solid line) defines the boundary for the maximum superjunction effect. The curves represent a set of points (N,w) ensuring the same E_th_ and the same blocking voltage V_BSJ,_ depending on the assumed thickness m of the SJM area only.

The above consideration proves that, generally, there are no theoretical obstacles to obtaining the superjunction diode with an unlimited breakdown voltage defined by (8) and (9). Its design must, however, adhere to several restrictions that ensure the presence of FFD areas in the pillars and their proper development under reverse bias conditions:

- In each pillar, the product N,w must correspond to a point inside the SJM region in Figure 9 to allow FFD areas to be built in.
- The junction planes separating pillars must be parallel to ensure the flatness of the electric field inside the FFD areas.
- To ensure the simultaneous development of the identical reverse voltage drop in the separated pillars, the magnitude of the electric field strength on the junction plane must be the same. It also concerns the threshold electric field E_thn_, which, taking advantage of (13), leads to the restriction:

(14) $$N_{D}w_{n}=N_{A}w_{p}$$

Let us notice that according to (8) and (9), the superjunction effect is more efficient when the threshold electric field E_th_ is small, which requires appropriately low-doped and narrow n- and p-pillars, to obtain the device length L and, following it, R_ON_ as small as possible.

#### 2.2.2. Forward Bias Conditions

**Figure 10:**
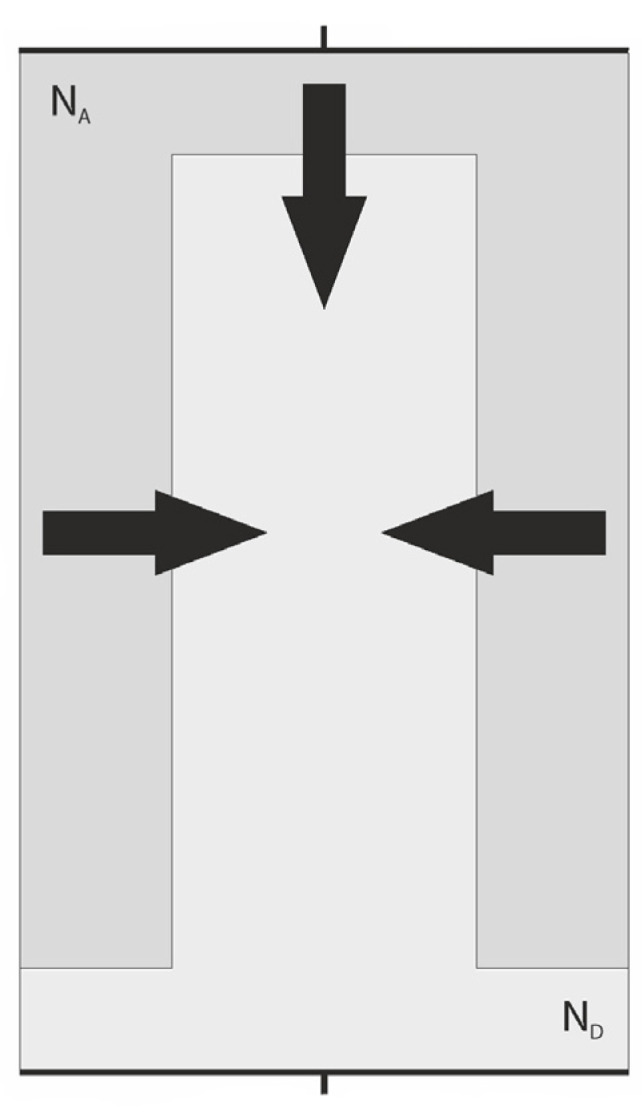
A section of the superjunction, with arrows indicating the directions of minority currents injected into the n-pillar—hole current at forward bias conditions.

The forward-biased superjunction in Figure 7 can be considered as a parallel set of pillars with planar p-n junctions connected by vertical back p-n junctions limiting the pillars on both sides. It implicates the 2D character of current flow in the device since the vertical and lateral carrier injection occurs. Such a situation is shown in Figure 10 in the case of one n-pillar only, where the arrows indicate the hole injection. The holes injected by vertical junctions, as the excess minority ones, increase the total carrier concentration, causing the modulation of n-pillar conductance that noticeably increases when the concentration of injected holes is sufficiently large. It is accompanied by a decrease in voltage drop along the n-pillar.

**Figure 11:**
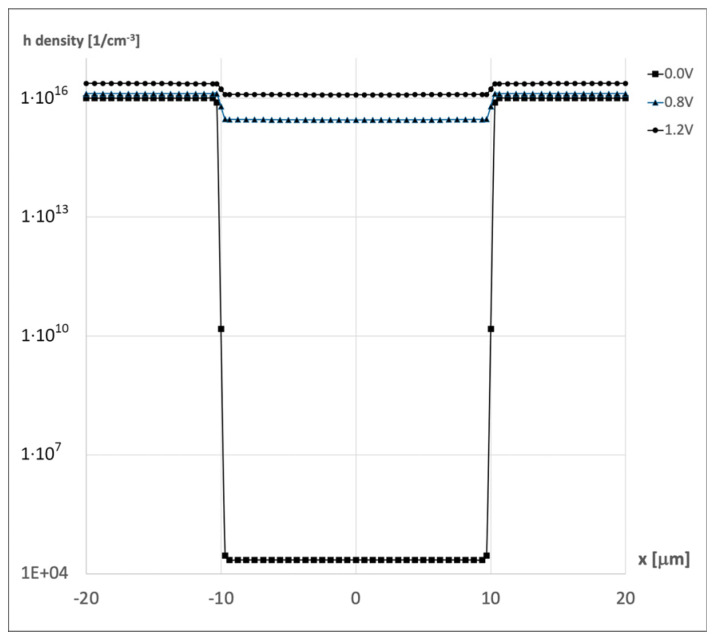
Concentration of excess carriers along the Y–Y axis in the n-type pillar—V_AC_ = 0 V; 0.8 V; 1.2 V.

The above situation is illustrated by Figure 11. It presents the result of numerical simulation [26] showing the distribution of injected holes in the n-pillar when the n-pillar has the same doping as the p-pillar (N_A_ = N_D_ = 1·10^16^ cm^−3^).

It can therefore be stated that in superjunction devices, the modulation of the on-resistance R_ON_ occurs in the conducting state. Thanks to this modulation effect, the resistance decreases as the forward voltage increases.

### 2.3. Superjunction CoolMOS™ Transistor

**Figure 12:**
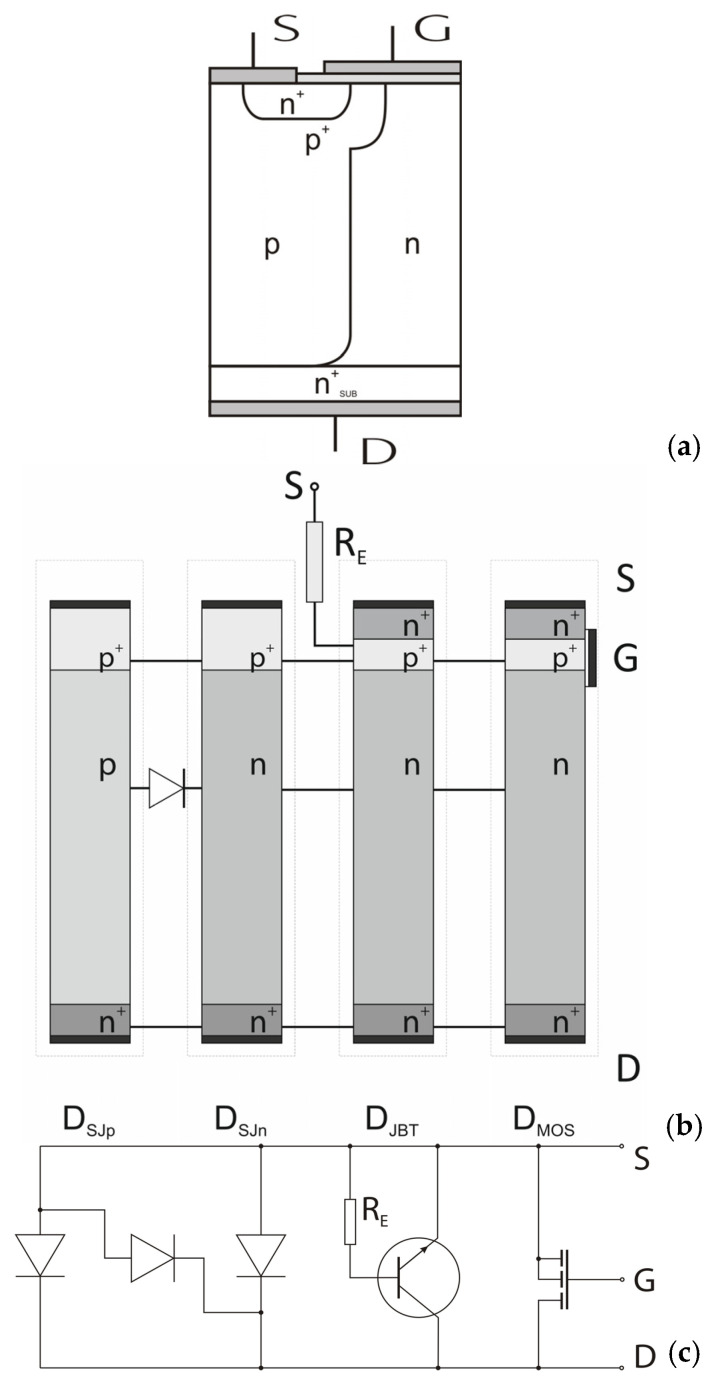
Elementary CoolMOS™ cell: sketch of elementary cell (**a**), autonomous section distinguished in the cell (**b**), and equivalent circuit of the cell (**c**).

In the CoolMOS™ structure depicted in Figure 1, one can distinguish a bipolar high voltage rectifying superjunction diode and a unipolar lateral MOS transistor that is connected in series with the n-pillar of the diode, as it is shown in the equivalent circuit sketched in Figure 12. It covers four autonomous sections extracted from the p- and n-pillars, which are connected in parallel and represent different paths for the drain-source current flow.

The sections D_SJn_ and D_SJp_ represent these parts of p- and n-pillars that create the p-pillar and n-pillar diodes introduced directly between S and D contacts. They are permanently reverse-biased, and their breakdown voltage is determined by (8) and (9). The section D_MOS_ covers the control MOS switch, allowing direct connection of the n^+^ source island and the n-pillar of the superjunction diode. When the switch is off, its breakdown voltage is determined by (9) if the channel length is sufficiently large. The Section D_JBT_ covers the parasitic bipolar transistor whose collector layer n is also the n-pillar layer, whereas the base p^+^ is connected with the source contact, both through the emitter layer n+ and the resistor R_E_ that reduces the effective emitter current. The reverse-biased collector junction keeps the section in a blocking state, but its reverse current is enlarged by the additional electron current injected from the n^+^ emitter. As a result, the density of electrons in the SCR layers of the collector junction essentially increases, and the avalanche breakdown develops at the lower electrical field strength E_critC_ = αE_crit_, where α is in the range 0.5 ÷ 1 and depends on the details of the section D_JBT_ design, i.e., the R_E_ value. It leads to the lower breakdown voltage:

(15) $$V_{B_{n}C}=\frac{\epsilon E_{{crit}_{C}}^{2}}{2q}\left(\frac{1}{N_{D}}+\frac{1}{N_{A}}\right)+\left(E_{{crit}_{C}}-E_{{th}_{n}}\right)m_{n}$$

On the basis of the above considerations, one can theoretically evaluate the basic relation R_ON_A versus V_B_ for the CoolMOS™ design shown in Figure 2. For this goal, the following assumptions have been taken into account:

- The CoolMOS™ forward current flows in the section D_MOS_ only, which reduces the effective area of current flow to the n-pillar;
- The CoolMOS™ breakdown voltage is limited to its lowest magnitude occurring in the section D_JBT_ and is determined by Relation (15);
- The first component of Relation (15) represents the breakdown of the planar p-n junction and is limited by the E_crit_, whereas the second one represents the superjunction effect and has no physical limits, and it is decisive mainly for the final breakdown voltage of CoolMOS™. Therefore, for simplicity, the Relation (15) can be reduced to the second component only:

(16) $$V_{B}=\left(\alpha E_{{crit}_{C}}-E_{{th}_{n}}\right)m_{n}$$

The effective CoolMOS™ on-resistance depends on the area of the n-pillar only and its magnitude, recalculated with respect to the device area A, is equal:

(17) $$R_{ON}A=\frac{m_{n}}{q\mu_{n}N_{D}}\frac{w_{n}+w_{p}}{w_{n}}$$

and, taking into account the Relation (5) rearranged for n-pillar, it can be rewritten in the form:

(18) $$R_{ON}A=\frac{m_{n}\left(w_{n}+w_{p}\right)}{2\epsilon \mu_{n}E_{{th}_{n}}}$$

and considering (16), we can obtain the desired relation:

(19) $$R_{ON}A=\frac{V_{B}\left(w_{n}+w_{p}\right)}{2\epsilon \mu_{n}E_{{th}_{n}}\left(\alpha E_{{crit}_{C}}-E_{{th}_{n}}\right)}$$

It is a linear dependence corresponding to the straight line in Figure 2, but it slightly differs from the relation presented in [2]. Its slope depends on the CoolMOS™ design parameters and can be both small or very large, which means that the relation between the lines for standard VDMOS and CoolMOS™ is not so unambiguous as it is shown in Figure 2. In some cases, the evaluated CoolMOS™ on-resistance may be even larger than the VDMOS one. To obtain low on-resistance, it is desired to keep a low magnitude of pillar widths and the threshold electric field strength E_thn_, as well as a large coefficient α.

## 3. Discussion

CoolMOS™ has been considered a device to overcome the high-voltage limitations of unipolar power devices. Although theoretical considerations support this possibility, practical results have not confirmed it so far. This expectation appears to have been overly optimistic. The present analysis is devoted to identifying these limitations.

Until now, the run of physical phenomena inside superjunction structures has only been touched on in the literature. The theoretical considerations focus on achieving a deeper understanding of these phenomena and on identifying the physical limitations inherent in the superjunction concept. As a result, the first coherent presentation of the concept evolution, from its physical background to practical applications, has been created. Its idea arises from the conclusion that in the ideal p-i-n diode, its middle layer has no dominant dopants, and no space charge region can build up in it, which results in the constant value of the electric field strength E_m_ in the layer. This way, the diode can block any voltage without the risk of avalanche breakdown when the middle intrinsic layer is sufficiently large. The only unacceptable disadvantage of such a high voltage diode is that its on-resistance is very large due to the lack of dopants in the middle layer.

This disadvantage can be eliminated by the introduction of dopants into the middle layer while keeping the flat electrical field distribution at the same time. It is possible to take advantage of the p-n superjunction concept, as it is shown in Figure 7. The superjunction diode is created as a set of parallel connected p-ν-n and p-π-n diodes, creating the characteristic meander-like p-n junction covering the set of n- and p-type pillars by turns. In such a design, the flatness of the electric field in the anode–cathode direction is ensured by side junctions between the pillars. Its theoretical analysis indicated that the superjunction effect is not an inherent feature of SJ diode design guaranteed by the meander-like p-n junction. According to (10), the superjunction component in the breakdown voltage V_BSJ_ can only exist when the threshold electric field E_th_ defined by (13) is lower than the maximum electric field E_crit_ corresponding to the avalanche breakdown in the considered semiconductor. Since E_th_ depends on the product (Nw), a supporting tool for designers in the form of a diagram in Figure 9 has been proposed. In the domain (N,w), two areas have been distinguished: SJM—gathers the points (N,w) ensuring the design of diodes with superjunction effect, and MN—gathers the points corresponding to diodes working in normal mode only.

The carried-out analytical consideration also follows the conclusions:

- The superjunction diode constitutes a parallel connection of several p- and n-pillars with their own breakdown voltages.
- The pillars’ junction planes need to be parallel to ensure the electric field remains uniform throughout the FFD regions.
- The proper work of the SJ diode requires the symmetry in the pillars design that should fulfill Condition (14).

The CoolMOS™ takes advantage of the SJ idea and covers such a meander-like p-n junction that has been introduced to ensure high voltage capability, which means that any SJ limitations relate to it as well. The carried out theoretical investigations pointed out that, as it is shown in Figure 11, in the cell of CoolMOS™ structure, one can distinguish four elementary devices covering SJ elements, which are connected in parallel between S and D contacts. The section DMOS includes an n-pillar, and only it conducts the forward current. It means that at the symmetric design of SJ, the theoretical path of on-current flow is two times smaller than the area of the cell cross-section, which results in an increase in on-resistance. All the sections take part in the reverse voltage blocking, but the final breakdown voltage of their parallel connection is defined by the worst element. It is the section D_BJT,_ including the bipolar transistor, whose breakdown voltage V_BnC_ is limited by the resistor R_E_ and equal to αV_Bn_, where V_Bn_—the breakdown voltage of n-column SJ and α is a bipolar transistor coefficient in the range 0.5 ÷ 1, depending on the details of section D_JBT_ design. It means that the CoolMOS™ breakdown voltage can be twice as small as the breakdown voltage of the built-in SJ structure. It is also worth mentioning that according to (19), the relation R_ON_A(V_B_) is linear as in Figure 2, but its slope depends on the CoolMOS™ design parameters. It can be both small or very large, which means that the relation between the lines for standard VDMOS and CoolMOS™ is not so unambiguous as it is shown in Figure 2.

## 4. Conclusions

Theoretical considerations indicate that, although CoolMOS™ is designed to alleviate high-voltage limitations in unipolar power devices, conceptual constraints prevent the realization of an ideal device without compromises. The introduction of superjunction elements increases breakdown voltage (V_B_) but also affects the on-state resistance (R_ON_), mainly due to the reduced effective current path in the n-pillars and the presence of parasitic effects. Performance depends critically on the symmetry and proper design of pillar dimensions, as well as doping distribution and correct junction alignment. The breakdown voltage is limited by the weakest element in parallel-connected superjunction structures, including the bipolar transistor section. Achieving an optimal balance between low on-resistance and high breakdown voltage requires careful consideration of the theoretical limitations in both the superjunction and MOS control parts, emphasizing the need for thorough device design.

## Data Availability

The original contributions presented in this study are included in the article. Further inquiries can be directed to the corresponding author.

## Abbreviations

The following abbreviations and symbols are used in this manuscript:

Abbreviations	
SJ	SuperJunction
SJM	SuperJunction Mode
NM	Normal Mode
N_A_, N_D_	doping concentration in the p- and n-layer
E, E_crit_, E_th_	electric field, critical electric field, threshold electric field
V_SJ_	reverse voltage drops in superjunction
V_B_, R_ON_	breakdown voltage, on-resistance
w_p_, w_n_	a and n column width
Symbols	
α	bipolar transistor coefficient
γ	superjunction coefficient
